# Living in two homes-a Swedish national survey of wellbeing in 12 and 15 year olds with joint physical custody

**DOI:** 10.1186/1471-2458-13-868

**Published:** 2013-09-22

**Authors:** Malin Bergström, Bitte Modin, Emma Fransson, Luis Rajmil, Marie Berlin, Per A Gustafsson, Anders Hjern

**Affiliations:** 1 Centre for Health Equity Studies (CHESS), Stockholm University/ Karolinska Institute, 106 91 Stockholm, Sweden; 2 Clinical Epidemiology, Department of Medicine, Karolinska Institutet, 171 77 Stockholm, Sweden; 3 Catalan Agency for Health Information, Assessment and Quality, and IMIM Hospital del Mar Research Institute, Barcelona, Spain; 4 National Board of Health and Welfare, 106 30 Stockholm, Sweden; 5 Department of Sociology, Stockholm University, 106 91 Stockholm, Sweden; 6 Child and Adolescent Psychiatry, Department of Clinical and Experimental Medicine, Linköping University, 581 85 Linköping, Sweden

**Keywords:** Alternate residency, Divorce, Joint physical custody, KIDSCREEN, Parental separation, Wellbeing

## Abstract

**Background:**

The practice of joint physical custody, where children spend equal time in each parent’s home after they separate, is increasing in many countries. It is particularly common in Sweden, where this custody arrangement applies to 30 per cent of children with separated parents. The aim of this study was to examine children’s health-related quality of life after parental separation, by comparing children living with both parents in nuclear families to those living in joint physical custody and other forms of domestic arrangements.

**Methods:**

Data from a national Swedish classroom study of 164,580 children aged 12 and 15-years-old were analysed by two-level linear regression modelling. Z-scores were used to equalise scales for ten dimensions of wellbeing from the KIDSCREEN-52 and the KIDSCREEN-10 Index and analysed for children in joint physical custody in comparison with children living in nuclear families and mostly or only with one parent.

**Results:**

Living in a nuclear family was positively associated with almost all aspects of wellbeing in comparison to children with separated parents. Children in joint physical custody experienced more positive outcomes, in terms of subjective wellbeing, family life and peer relations, than children living mostly or only with one parent. For the 12-year-olds, beta coefficients for moods and emotions ranged from −0.20 to −0.33 and peer relations from −0.11 to −0.20 for children in joint physical custody and living mostly or only with one parent. The corresponding estimates for the 15-year-olds varied from −0.08 to −0.28 and from −0.03 to −0.13 on these subscales. The 15-year-olds in joint physical custody were more likely than the 12-year-olds to report similar wellbeing levels on most outcomes to the children in nuclear families.

**Conclusions:**

Children who spent equal time living with both parents after a separation reported better wellbeing than children in predominantly single parent care. This was particularly true for the 15-year-olds, while the reported wellbeing of 12-years-olds was less satisfactory. There is a need for further studies that can account for the pre and post separation context of individual families and the wellbeing of younger age groups in joint physical custody.

## Background

The traditional living arrangement in Western countries after a parental separation has been maternal single care and studies show that children’s contact with their father in these situations can deteriorate or even disappear over time [1,2]. However, during the last few decades, this pattern has changed in many countries, so that more children maintain relationships with both parents and spend more time with their fathers after a family dissolution [3]. An increasing number of children are in joint physical custody (JPC), which refers to a practice where the child spends equal or substantial amounts of time in the parents’ respective homes [2,4]. In Sweden, the rise in JPC has been dramatic, rising from about one per cent of children in post separation families in the mid 1980's to nearly 30 per cent in recent years [2].

In addition to parental gender equality, the increasing number of women in paid employment and changes to family law have been suggested as plausible reasons behind the trend towards JPC among separating parents [3,4]. Since 1998, joint custody has been the default legal practice in Sweden if the parents are married and the courts have had the power to decide to impose a JPC arrangement since that date [5]. The Swedish frequency of JPC is high in comparison with other countries [4], but the practice is also increasing in other Western countries. It now accounts for about 16 per cent of the children with separated parents in Australia [6], nine to 15 per cent in Canada [7], nine to 17 per cent in the UK [3] and around 20 per cent in the USA, Denmark and The Netherlands [8-10].

In Sweden, about half a million children (25 per cent) have parents who do not live together [2]. This figure is in line with that of other European countries, as well as the USA [11]. The risk of separation is particularly high among couples who are young or have a low level of education [2] and the family’s socioeconomic situation also influences children’s living arrangements if the parents move apart [3,9,12,13]. It is more common for parents in families with JPC to have higher levels of education [3,9,10,12], dual incomes [3,9,12] and low levels of marital or post separation conflict [9,12,14-17] than single-custody families. They are also less likely to be migrants [10,18]. But parents with high conflict levels may still choose JPC for their children. In Sweden, as well as in countries such as Australia, courts, counsellors and mediators may favour JPC even if one parent prefers single custody [14,19]. It has been estimated that around 14 per cent of Swedish parents who separate are involved in conflict about their children’s custody and housing [19].

The risks of emotional problems, social maladjustment and low levels of wellbeing are higher in children with separated parents than those in nuclear families [20-22]. Some studies indicate that boys may experience more negative consequences than girls, at least when it comes to short-term, externalised behaviour problems [23,24]. This could be due to them losing the gender role model provided by their father [25]. However, other studies have found no gender differences in children’s adjustment and wellbeing after parental separation [26] or more negative experiences in girls [27]. Children’s health and wellbeing is affected by a number of factors when their parents separate. These include economic deprivation, loss of parental supervision or social networks and the parent’s psychological abilities to solve conflicts, tackle practical challenges and sustain engagement in parenthood [28,29].

The consequences of JPC for children’s health, development and wellbeing have been debated [14,30]. Some authors suggest that on-going access to the households and resources of both parents may reduce the economic stress and disadvantages that parental separation may otherwise impose on a child. It has been argued that continuing everyday contact with both parents enables them to feel close to the child and in touch with their development and relieves the child from the potential sense of loss of one parent [10,15-17]. Others emphasise the negative aspects of JPC, like the burden of moving between houses, lack of stability in parenting and the home environment, a sense of alienation and a risk of increased exposure to parental conflict [14,31].

In conclusion, research has identified post-separation factors that are detrimental to children’s wellbeing, but draw different conclusions about how JPC and other living arrangements may affect children’s health and wellbeing. Much still remains unclear about the benefits and drawbacks of the comparatively new and increasing practice of JPC and how these vary by age and gender.

In this study we took advantage of the comparatively high occurrence of JPC in Sweden to compare health related quality of life (HQL) for children in nuclear families with JPC and other forms of living arrangements after parental separation. We did this by using data from a national classroom survey of 12 and 15-year-olds.

## Methods

### Data source

We used data from a 2009 national survey of wellbeing and mental health in pupils in grade six (mean age 12 years) and grade nine (mean age 15 years), conducted by Statistics Sweden under the mandate of The Swedish National Institute of Public Health [32]. The Swedish National Board of Health and Welfare gave us permission to use the data. A total of 207,700 pupils were eligible to take part in the survey and 172,391 (83 per cent) were at school when the survey was conducted and agreed to participate. We included the 164,580 (79 per cent) who had completed the KIDSCREEN instrument, answered the questions on living arrangements and went to a school with at least ten pupils in their grade.

### Outcome measures

We used eleven dimensions of health related quality of life (HQL) from the KIDSCREEN-52 scale [33] and the KIDSCREEN-10 Index. KIDSCREEN was developed by research groups from 13 European countries to measure wellbeing in children and adolescents aged eight to 18- years-of-age [34]. HQL is a multidimensional construct that includes an individual’s perception of his/her emotional, physical, social, mental and behavioural wellbeing. The instrument includes the subscales of:

- Physical health (*Have you felt fit and well?,* five items)

- Psychological wellbeing (*Have you felt satisfied with your life?,* six items)

- Moods and emotions (*Have you felt that you do everything badly?*, seven items)

- Self perception (*Have you been happy with the way you are?,* five items)

- Autonomy (*Have you been able to choose what to do in your free time?,* five items)

- Parent relations (*Have you been able talk to your parent(s) when you wanted to?,* six items)

- Material resources (*Have you had enough money to do the same things as your friends?,* three items)

- Peer relations (*Have you been able to rely on your friends?,* six items)

- School satisfaction (*Have you enjoyed going to school?,* six items) and

- Social acceptance and bullying (*Have you been afraid of other girls and boys?*, three items)

The questions concern the respondent’s experiences from the previous week with response options assessing either intensity (*not at all, slightly, moderately, very or extremely*) or frequency (*never, seldom, sometimes, often or always*). High scores indicate greater wellbeing.

Rasch analysis was used for the respective dimensions [35] and the scores were transformed to Z-values with mean value 0 and standard deviation 1 [36]. This instrument has shown acceptable reliability and validity [35].

### Categorical variable

The children chose from the following categories in the survey to describe their family arrangements: nuclear family (“always together with both mother and father”); JPC (“approximately equal time with mother and father, for example one week with mother and the second week with father”); *mostly with one parent* (“mostly with mother, sometimes with father” or “mostly with father, sometimes with mother”) and *only with one parent* (“only with mother” or “only with father”). We merged the gender specific alternatives in the “*mostly*” and “*only*” categories as preliminary analysis showed that parental gender had less importance in relation to the outcomes than the living arrangements.

### Covariates

The covariates gender, grade and country of origin were obtained from the questionnaire. Data on the parent’s level of school education, domicile (large city, other city or small town and rural) and migrant origin were obtained from the National SIRIS database [37] and linked to the data set through the school identification number. Domicile was based on the categories provided by the Swedish Association of Local Authorities and Regions.

### Statistical analysis

We performed multivariate two-level analysis, employing a random intercept model and stratified by grade [38]. Linear multivariate two-level analysis were used to calculate beta coefficients on the eleven standardised scales for the four family types, with the nuclear family as the reference category in the primary analysis. A separate analysis was carried out to compare differences between children in JPC and those living mostly or only with one parent. This analysis included the three forms of post separation family types but living “*mostly*” and “*only*” with one parent were collapsed into one category. Individual covariates were gender and country of origin (Swedish versus foreign born). Three further covariates domicile (large city/other city/small town plus rural), percentage of parents with post-secondary education and immigrant origin, were analysed at the school level. The statistical package used for the multilevel analyses was MLwiN 2.28 [39]. Adjusting for covariates had little or no effect on the risk estimates and therefore only the fully adjusted model is shown in Table 1 (complete analysis available on request). Interaction analyses demonstrated differences in the effects of JPC on seven measures of wellbeing between the 12 and 15-year-olds, which led to the decision to perform the analysis separately in the two age groups.

**Table 1:** Sociodemographic characteristics in relation to living arrangements, in numbers and percentages, N = 164,580

		**Nuclear family**		**JPC equally much with both parents**		**Mostly with one parent**		**Only with one parent**	
		**n = 112,778**		**n = 17,350**		**n = 12,800**		**n = 21,652**	
**Individual characteristics**		n	%	n	%	n	%	n	%
**Gender**	Boy	56,433	69.1	8923	10.9	6218	7.6	10,077	12.3
	Girl	54,942	68.0	8199	10.1	6403	7.9	11,242	13.9
**Grade**	Six (12 years)	34,933	71.8	5637	11.6	3527	7.3	4535	9.3
	Nine (15 years)	43,247	67.3	6531	10.2	5140	8.0	9326	14.5
**Origin**	Swedish born	102,824	68.4	16,866	11.2	12,164	8.1	18,474	12.3
	Foreign born	9954	69.8	484	3.4	636	4.5	3178	22.3
**Resident parent’s gender**	Mother					7165	82.7	11,544	83.3
	Father					1502	17.3	2317	16.7
**School level characteristics**									
**Domicile**	Large city	22,498	69.3	5963	11.5	2672	8.2	4349	13.4
	Other city	54,777	68.4	8436	10.5	6490	8.1	10,363	12.9
	Rural	35,396	68.2	2935	9.0	3630	7.0	6926	15.8
**Proportion of parents with migrant background**	0-10%	55,603	69.7	8919	11.2	6323	7.9	8893	11.2
	11-50%	46,699	66.9	7578	10.9	5594	8.0	9932	14.2
	>50%	7853	69.5	491	4.3	619	5.5	2337	20.7
**Proportion of parents with post-secondary education**	0-40%	34,980	67.6	4275	8.3	4073	7.9	8444	16.3
	41-60%	46,874	68.2	7552	11.0	5583	8.1	8725	12.7
	61-100%	28,301	70.2	5161	12.8	2880	7.1	3993	9.9

## Results

### Descriptive statistics

Table 1 shows that 10.4 per cent of the children lived in a JPC arrangement, 7.7 per cent *mostly* one parent, 13.1 per cent *only* with one parent and 68.5 per cent in nuclear families. These numbers show that the majority of Swedish 12 and 15-year-olds live, at least partly, with both of their parents. There were small differences in living arrangements in relation to the children’s sex and domicile, but a larger proportion of the 15 year-olds lived with *only* one parent than the 12-year-olds. Foreign-born children had similar rates of separated parents compared to Swedish born children, but living with *only* one parent, predominantly the mother, was the most common post separation living arrangement in this group. The frequency of JPC also varied at the school level when it came to the proportion of children with migrant backgrounds. JPC and living with *only* one parent were equally common in schools where 10 per cent or less of the children had migrant backgrounds. Where more than half of the pupils had migrant backgrounds, living with *only* one parent was more than four times as common as JPC. The frequency of JPC also varied with parental education. More JPC children were in schools with higher proportions of parents with post-secondary education. All these differences were statistically significant at the <0.001 level.

### Wellbeing

Regardless of age, children in nuclear families reported better wellbeing than children with separated parents (Table 2). However, the 15-year-olds in JPC and nuclear families had similar levels of wellbeing on the autonomy, peer relations and social acceptance scales. Also the 15-year-olds who lived with *only* one parent reported similar wellbeing on autonomy, as did those living *mostly* with one parent on social acceptance (bullying), compared with nuclear families.

**Table 2:** Z-transformed beta-estimates of living arrangements in a two-level linear regression analysis of various dimensions of health related quality of life (HQL) measured with KIDSCREEN

		**12 year olds**	**15 year olds**
a. **Kidscreen-10**	Nuclear family	0	0
	Joint physical custody^1^	-0.24***	-0.11***
	Lives mostly with one parent	-0.32***	-0.23**
	Lives only with one parent	-0.25***	-0.24***
b. **Physical health**	Nuclear family	0	0
	Joint physical custody^1^	-0.22***	-0.20***
	Lives mostly with one parent	-0.33***	-0.29***
	Lives only with one parent	-0.30***	-0.34***
c. **Psychological well-being**	Nuclear family	0	0
	Joint physical custody^1^	-0.25***	-0.13***
	Lives mostly with one parent	-0.37***	-0.25***
	Lives only with one parent	-0.35***	-0.33***
d. **Moods and Emotions**	Nuclear family	0	0
	Joint physical custody^1^	-0.20***	-0.08**
	Lives mostly with one parent	-0.32***	-0.21***
	Lives only with one parent	-0.33***	-0.28***
e**. Self Perception**	Nuclear family	0	0
	Joint physical custody^1^	-0.22***	-0.09***
	Lives mostly with one parent	-0.27***	-0.19***
	Lives only with one parent	-0.25***	-0.23***
f**. Autonomy**	Nuclear family	0	0
	Joint physical custody^1^	-0.13***	0.00
	Lives mostly with one parent	-0.19***	-0.08**
	Lives only with one parent	-0.12***	-0.03
g. **Parent relations**	Nuclear family	0	0
	Joint physical custody^1^	-0.23***	-0.16***
	Lives mostly with one parent	-0.39***	-0.35***
	Lives only with one parent	-0.35***	-0.39***
h. **Material resources**	Nuclear family	0	0
	Joint physical custody^1^	-0.20***	-0.16***
	Lives mostly with one parent	-0.34***	-0.31***
	Lives only with one parent	-0.41***	-0.45***
i. **Peer relations**	Nuclear family	0	0
	Joint physical custody^1^	-0.11***	-0.03
	Lives mostly with one parent	-0.20***	-0.08**
	Lives only with one parent	-0.20***	-0.13***
j**. School satisfaction**	Nuclear family	0	0
	Joint physical custody^1^	-0.25***	-0.14***
	Lives mostly with one parent	-0.32***	-0.25***
	Lives only with one parent	-0.28***	-0.30***
k. **Social acceptance**	Nuclear family	0	0
	Joint physical custody^1^	0.05**	0.02
	Lives mostly with one parent	0.16***	0.02
	Lives only with one parent	0.22***	0.07**

*** = p < 0.001, ** = p < 0.01. The estimates are adjusted for gender and origin on the individual level and for domicile and proportion of pupils with parents with post-secondary education and with foreign background on the school level.

¹equally much with both parents.

Wellbeing among both the 12 and 15-year-olds in JPC was better than for the other two forms of post separation living arrangements collapsed into one category, p < 0.05-p < 0.001, for all the subscales, with the exception of autonomy, self-perception and school satisfaction for the 12-year-olds and self perception for the 15-year-olds.

We performed interaction analyses to investigate the differences between the 12 and 15-year-olds in JPC and found that the 12-year-olds reported a lower relative wellbeing than the 15-year-olds on seven of the eleven subscales. The wellbeing of the 15-year-olds shared greater similarities with the nuclear families than the younger age category when it came to the comprehensive Kidscreen-10 measures, emotional wellbeing (psychological wellbeing, moods and emotions and self perception), peer and parent relations and school satisfaction. However, the 12-year-olds in JPC still tended to report better wellbeing on these scales than 12-year-olds with other post separation living arrangements. The interaction analyses showed no differences between boys and girls on the effects of JPC on wellbeing.

## Discussion and conclusions

In this study, based on a national classroom survey of nearly 165,000 Swedish 12 and 15-year-olds, children with separated parents reported lower levels of wellbeing than children in nuclear families. The degree of wellbeing, however, varied in relation to living arrangements. Overall, children in JPC arrangements reported more positive wellbeing than those living *mostly* or *only* with one parent. Furthermore, the 12-year-olds in JPC reported lower levels of wellbeing to the 15-year-olds in the same living arrangement.

The finding that children with separated parents report lower wellbeing than children with married or cohabiting parents is in line with previous Swedish [18,40] and international research [12,23]. However, earlier findings show less agreement about how children in JPC fare.

We found that both the 12 and 15-year-olds in JPC reported better wellbeing on most of the measured aspects than those living *mostly* or *only* with one parent. This finding is in line with a recent Swedish publication on subjective health complaints and wellbeing in children of 11 to 15- years-of-age [18]. In addition, another Swedish study of 15-year-olds did not find any increased risk of mental distress or victimisation for teenagers in JPC compared to those in nuclear families [40].

In the international literature, a meta-analysis of studies from the 1980s and 1990s concluded that children in JPC were as adjusted as those in nuclear families [17]. More recent publications have found that children living in JPC reported better emotional wellbeing and social adjustment than children living with one parent [9,10,15,16,22]. Children in JPC have been found to have closer and more positive contact with their father than children in single care [10,11,16]. This, in turn, has been shown to be related to positive health outcomes [41]. A large-scale study of children from 36 countries did, however, find similar life satisfaction in children with JPC and single care after adjustment for family affluence [21].

Our finding that 12-year-olds reported lower levels of wellbeing to 15-year-olds indicates that living in two homes poses different challenges for children of different ages. It is possible that the 15-year-olds had greater opportunities to influence their living arrangements than the 12-year-olds. We can also speculate that the lower levels of wellbeing among the younger children could possibly be related to the greater significance of the parents and family in the younger children’s psychological and social life. In general, the emancipation process of adolescence has not started at the age of 12 [42]. It is likely to have a greater impact on the 15-year-olds, who are psychologically in the middle of forming their own identity, friends and social networks outside of the family. Adolescents are also more prone to risk behaviours than younger peers [42] and, as a result, they may benefit from living with two parents who set boundaries. Thus, it may reasonable to assume that because the 15-year-olds are more independent, they are better equipped to cope with the challenge of having two alternating homes.

Our findings stress the need to evaluate living arrangements after parental separation in relation to children’s psychological development. This is particularly important because studies from several countries show that JPC is most common for children in middle childhood [2,9,12,43]. In some studies on the impact of parental separation on children’s wellbeing, the relevance of the children’s age has been included, for example in Lansford’s analyses, which suggested that children in middle childhood experienced more emotional problems and that teenagers had more problems with their school performance [44]. In other studies, such as a meta-analysis on divorce and parental conflict, the author underlines the lack of information on the children’s ages in studies [20]. Other studies of children’s wellbeing, adjustment or health in relation to JPC do not report or explore age differences in children in middle childhood and adolescence either [10,11,21,30]. However, the practice of JPC has been questioned for very young children and it has been argued that toddlers can be expected to have greater difficulties in coping with alternating homes than older children [14,45].

### Strengths and limitations

The large national population that we analysed in this study allows us to draw conclusions on the entire population of 12 and 15-year-olds, in contrast to most previous studies of JPC that have suffered from small samples with considerable problems of attrition [17].

A further strength is the categorisation of post separation living arrangements, distinguishing the children who live *mostly* with one parent from those in JPC. This is more effective in highlighting the subtle differences than many other studies, where JPC may include children living for 30 per cent or less of the time with one parent [9,14,15] or even families with joint legal custody and primary residency with one parent [17]. This study is based on a precise definition of JPC, in that the JPC children spent equal time living with both the mother and father, for example one week with the mother and then the next week with the father. The wording of the question implies that the children who chose this response actually spend 50% of their time in each parent’s home.

The population sample also ensured that we included families with different background characteristics that may influence children’s wellbeing. On the other hand, our data is limited when it comes to the contextual variables of the individual families, such as socioeconomic situation or level of parental conflict. As a result, we have no knowledge about how these factors affect the children in the different living arrangements. This is an important limitation, because such factors may be associated with children’s post separation living arrangements and also directly affect the children’s wellbeing [21,28,29].

There are also several important family determinants of wellbeing in children associated with parental separation. For example, separated parents are more likely to have mental health problems and poor finances and the separation is often preceded by a shorter or longer period of marital discord [2,46]. Also, factors such as parent’s level of conflict and ability to cooperate after the separation are known to impact on children’s wellbeing [9,47]. Information about these circumstances, and the time that had elapsed since the parents separated, were not available in this study. The cross-sectional design is another limitation in this respect. Therefore, the broad pattern of children’s wellbeing in relation to living arrangements presented in this study need to be confirmed by longitudinal cohort studies that can evaluate living arrangements in relation to this pre- and post separation context.

### Implications

Both the 12 and 15-year-olds in JPC in this study reported higher levels of wellbeing than children in predominantly single parent care after parental separation. However the 12-year-olds report a less satisfactory wellbeing than the 15-year-olds, suggesting that JPC may pose different challenges for children of different ages. Further studies that look at the pre and post separation context and younger age groups are needed to inform policies covering custody after parental separation.

## Abbreviations

JPC: Joint physical custody; HQL: Health related quality of life.

## Competing interests

The authors declare that they have no competing interests.

## Authors’ contributions

MBe conceived the study, participated in the design and drafted the manuscript. BM provided expertise regarding the data source, interpretation of the data and the statistical analysis. EF participated in the design of the study and interpretation of the data. LR provided methodological expertise regarding the outcome measures and participated in the analysis. MB provided expertise regarding demography and the data source and participated in the interpretation of the data. PAG provided expertise regarding child psychiatry and participated in the interpretation of the data. AH participated in the design of the study, provided expertise regarding the data source, performed the statistical analyses and interpretation of the data and helped to draft the manuscript. All authors read and approved the final manuscript.

## Pre-publication history

The pre-publication history for this paper can be accessed here:

http://www.biomedcentral.com/1471-2458/13/868/prepub
